# Repentance

**Published:** 1890-03-08

**Authors:** 


### Words of Consolation.

### REPENTANCE.

*For Reading to the Sick.*

God gives us many opportunities during our lives of recalling our past sins, and realizing how we stand in His sight. Let us, therefore, take these hours of sickness or partial recovery to think over our own particular shortcomings, and find out whether we are content to live afar from God, or have already gone to that Fountain which alone cleanses from sin. For though in the eyes of men there are various degrees of sinning, yet in the sight of the Almighty all men are alike sinners till they have been washed in Christ's atoning blood.

We will first ask ourselves, then, have we spent our lives hitherto in the service of God or of the world? By serving the world is not here meant the sins of the flesh, such as drunkenness and gluttony, murder, adultery, and theft. Those who give way to such excesses are sometimes suddenly stopped in their headlong course of wickedness by the interposition of God's mercy and receiving forgiveness from Christ, notwithstanding the greatness and depth of their sins, their repentance and love and sorrow are in proportion to the fullness of their pardon. But we are now thinking of such as have spent an ordinary life amidst the temptations which beset the path of all Christians. Have we looked upon ourselves as safe, because we have not been actively wicked? Do we go to worship God in His house only when it is convenient, or we feel inclined, instead of looking on it as a duty never to be neglected? Do we find ourselves too tired or sleepy at night to pray to the Almighty, or go off to our daily tasks without thanking Him for keeping us safely through the hours of darkness, and asking for His guidance through the coming day? In short, are we active and persevering in our own business, but listless and slothful in God's service? If so, we are but as dying men from whom life is fast ebbing away. We shall soon be past all hope dead in trespasses and sins, if we neglect the Master to whose service we have been vowed in baptism. Then, again, are we strictly honest, never using short weights or light measures? Do we speak the truth always and act the truth? Do we check the angry word, the hasty blow? Do we give the soft answer that turneth away wrath? These and many like questions we should ask ourselves, and repent now while there is time. God's judgments are hanging over the heads of all careless sinners, and may fall on them at any moment. "But who may abide the day of His coming, and who shall stand when He appeareth?" No man in his own strength. It is of the Lord's mercies alone that we shall not be consumed when He cometh to divide the wheat from the chaff, the good from the wicked, in the Day of Judgment. Let us not, then, abuse the goodness of God, who calls us daily to repentance; out of His endless pity He promises us forgiveness, if we truly turn to Him with all our hearts. "For though our sins be as scarlet they shall be white as snow; and though they be as purple they shall be made white as wool." Let us cast away from us all our sloth—our neglect of God's worship and His laws. Let us come to our Lord and Saviour, and He will give us new hearts, for why should we die? God willeth not the death of a sinner, but rather that he should be converted and live. "If any man sin," says St. John, "we have an advocate with the Father, Jesus Christ the righteous, and He is the propitiation for our sins." Let us not despise the Lord, who bought us.

